# Time-dependent LPS exposure commands MSC immunoplasticity through TLR4 activation leading to opposite therapeutic outcome in EAE

**DOI:** 10.1186/s13287-020-01840-2

**Published:** 2020-09-25

**Authors:** Mónica Kurte, Ana María Vega-Letter, Patricia Luz-Crawford, Farida Djouad, Danièle Noël, Maroun Khoury, Flavio Carrión

**Affiliations:** 1 Laboratorio de Inmunología Celular y Molecular, Centro de Investigación Biomédica, Universidad de Los Andes, Santiago, Chile; 2 Programa de Doctorado en Biomedicina, Facultad de Medicina, Universidad de los Andes, Santiago, Chile; 3 Cells for Cells, Regenero, Av. Álvaro del Portillo 12.455, Las Condes, Santiago, Chile; 4 Laboratory of Nano-Regenerative Medicine, Faculty of Medicine, Universidad de los Andes, Santiago, Chile; 5 IRMB, Univ Montpellier, INSERM, Montpellier, France; 6 CHU Montpellier, Montpellier, France; 7 Programa de Inmunología Traslacional, Facultad de Medicina, Clínica Alemana Universidad del Desarrollo, Av. Las Condes 12496 Lo Barnechea, Santiago, Chile

**Keywords:** MSCs, TLR4, LPS, Immunosuppression, Cell plasticity, Autoimmune diseases

## Abstract

**Background:**

Mesenchymal stem cells (MSCs) have been recognized for their regenerative and anti-inflammatory capacity which makes them very attractive to cell therapy, especially those ones to treat inflammatory and autoimmune disease. Two different immune-phenotypes have been described for MSCs depending on which Toll-like receptor (TLR) is activated. MSC1 is endowed with a pro-inflammatory phenotype following TLR4 activation with LPS. On the other hand, anti-inflammatory MSC2 is induced by the activation of TLR3 with Poly(I:C). High immunoplasticity of MSCs is a matter of concern in cell-based therapies. In this study, we investigated whether a single stimulus can induce both types of MSCs through a differential activation of TLR4 with LPS.

**Methods:**

MSCs were activated with LPS following a short exposure of 1-h (MSCs-LPS1h) or long-time exposure for 48 h (MSCs-LPS48h), and then, we evaluated the biological response in vitro, the immunosuppressive capacity of MSCs in vitro, and the therapeutic potential of MSCs in an experimental autoimmune encephalomyelitis (EAE) mouse model.

**Results:**

Our results showed that 1-h LPS exposure induced a MSC1 phenotype. Indeed, MSCs-LPS1h expressed low levels of NO/iNOS and decreased immunosuppressive capacity in vitro without therapeutic effect in the EAE model. In contrast, MSCs-LPS48h achieved a MSC2-like phenotype with significant increase in the immunosuppressive capacity on T cell proliferation in vitro, together with an improved in the therapeutic effect and higher Treg, compared to unstimulated MSCs. Furthermore, we determine through the MSCs-TLR4KO that the expression of TLR4 receptor is essential for MSCs’ suppressive activity since TLR4 deletion was associated with a diminished suppressive effect in vitro and a loss of therapeutic effect in vivo.

**Conclusions:**

We demonstrate that MSCs display a high immunoplasticity commanded by a single stimulus, where LPS exposure time regulated the MSC suppressive effect leading into either an enhanced or an impairment therapeutic activity. Our results underscore the importance of phenotype conversion probably related to the TLR4 expression and activation, in the design of future clinical protocols to treat patients with inflammatory and autoimmune diseases.

## Introduction

Mesenchymal stem cells (MSCs) are adherent, undifferentiated, multipotent, and non-hematopoietic progenitor cells. MSCs have the potential to differentiate into mesodermal lineages including osteoblasts, chondrocytes, and adipocytes, among others [1, 2], promoting a great therapeutic value for regenerative medicine [3–5]. MSCs are also known for their ability to regulate all the different components of the immune system, especially for being able to modulate lymphocyte activity.

The immunomodulatory properties confer to MSCs are valuable therapeutic attributes for pro-inflammatory and autoimmune diseases [6–8]. In different preclinical models such as collagen-induced arthritis (CIA) [9], ulcerative colitis [10], and experimental autoimmune encephalomyelitis (EAE) [11–13], an improvement of the symptoms and prognosis of the disease were observed after treatment with MSCs. The induction of MSCs immunosuppressive functions is mainly provided by the stimulation with pro-inflammatory cytokines such as IFNγ, TNF, and IL1β [14]. In addition, MSCs can be activated by different Pathogen-Associated Molecular Patterns (PAMPs), which correspond to molecules recognized by a set of receptors known as Pattern Recognition Receptors (PRRs), among which Toll-like receptors (TLRs) have been studied [15–17]. Despite TLRs are expressed mainly in antigen-presenting cells and their function is directly associated with the activation of the immune response, MSCs have been also described to express most of the different types of TLRs that modulate different functions like proliferation, differentiation, migration and immunosuppressive potential [18–21].

Regardless of these reports, there is no consensus on TLR activation and its impact on the immunomodulatory properties of MSCs. Different results have been obtained depending on the origin of the MSCs, type of stimulus, concentration, or the in vitro model used to evaluate their immunosuppressive properties.

In 2010, Waterman et al. defined two different phenotypes for human MSCs, depending on which TLRs were activated [22]. They described the MSC type 1 (MSC1), endowed with a pro-inflammatory phenotype, after TLR4 activation with LPS by 1 h. On the other hand, anti-inflammatory MSC type 2 (MSC2) phenotype is induced after the activation of TLR3 with Poly(I:C) by 1 h. Of note, other immune system cells, such as macrophages (M) and dendritic cells (DCs), have been described to adopt different phenotypes and functions according to the microenvironment they encountered. Thus, DCs may act as pro-inflammatory (DC1) or anti-inflammatory (DC2) [23, 24], as well as macrophage types 1 (M1) and 2 (M2) [25].

Published data from our laboratory showed that murine bone marrow-derived MSCs stimulated for 1 h with LPS (MSCs-LPS1h) exert a pro-inflammatory phenotype since they lose the ability to inhibit T cell proliferation and lose their therapeutically potential in EAE [26]. On the contrary, MSCs stimulated with Poly(I:C) for 1 h (MSCs-Poly1h) acquired an anti-inflammatory phenotype showing higher immunosuppressive capacity compared to control MSCs and decreased clinical score in EAE [26]. These results conclude that murine MSCs, similar to human MSCs, also display a dual phenotype, MSC1 or MSC2, depending on which TLRs are activated.

However, how exactly MSCs respond to the same inflammatory stimuli at different exposure time remains to be investigated. While in previous studies, two different stimulations (LPS or Poly(I:C)) were used to generate the two types of MSCs, in the present work, we investigated whether a stimulation with a single agent (LPS) could exert a dual effect on MSCs depending on variable activation of the TLR4. Thus, we propose that the induction of MSC1 or MSC2 phenotypes is achievable through the differential activation of TLR4, indicating their fundamental role in the induction of MSCs’ immunoregulatory properties and MSCs’ high plasticity, which should be addressed when used in inflammatory and autoimmune therapies. Here, we have demonstrated that MSCs’ response to different time point exposure to LPS significantly modulates their suppressive and therapeutic efficacy in the EAE murine model. This effect was mediated, at least in part by the TLR4 signaling pathway.

## Material and methods

### Animals

Female C57BL/6 mice, 8–14 weeks old, were obtained from the Central Animal Facility, Instituto de Salud Pública (ISP), Santiago, Chile. TLR4 knockout (B6.B10ScN-Tlr4<lps-del>/JthJ) were obtained from Jackson Laboratory, Bar Harbor, ME, USA. Animals were housed in a high barrier animal facility and received irradiated food (Picolab Mouse Diet, PAIS) and acidic water (pH 3.0) ad libitum. Experimental procedures and protocols were performed according to the US National Institute of Health Guide for the care and use of laboratory animals (NIH Publication No. 85-23, revised 1996) and were approved by the Bioethics Committee of the Universidad de los Andes and by Fondecyt Bioethics Advisory Committee, Chile.

### MSC isolation and culture

Murine bone marrow-derived MSCs were obtained from C57BL/6 wild type and TLR4 knockout (B6.B10ScN-Tlr4<lps-del>/JthJ) mice (For mice information see Supp. Fig. 1). Isolation and phenotypic and functional characterization of MSCs and MSCs-TLR4KO were performed as previously described [1, 26]. MSCs-TLR4KO phenotypic characterization and TLR4 expression were analyzed by PCR and flow cytometry (Supp. Fig. 2).

### Quantitative real-time PCR (qPCR)

Total RNA was isolated using Trizol reagent (Ambion, Life Technology) and treated with DNAse I (Fermentas, MA, USA). Two micrograms of DNAse-I-treated RNA was reverse transcribed using ImpromRT and random hexamers (Promega, WI, USA) in 30 μl total volume reaction, according to the manufacturer’s recommendations. PCR assay was performed using 2.5 μl of diluted cDNA (1:100 to 1:500) and 10 μl of primer-containing GoTaq MasterMix (Promega, WI, USA) 150 pmol each primer and analyzed using Mx3000P qRT-PCR system (Agilent Technologies). The following primers were used: sense: iNOS 5′-AGTTCTGCGCCTTTGCTCAT-3′ and antisense: 5′-AGTGAAGCGTTTCGGGATCT-3′; for IL-6, sense: 5′-CCTTCCTACCCCAATTTCCA-3′ and antisense: 5′-GGCATAACGCACTAGGTTTG-3′; for 18S, sense: 5′-ATCGCCAGTCGGCATCGTTTAT-3′ and antisense: 5′-GCCGCTAGAGGTGAAATTCTTGGA-3′. Expression level of transcripts was normalized to 18S mRNA levels (normalizer) and to control healthy mice (control) according to the formula 2−Δ(ΔCT) [27].

### Flow cytometry

Surface staining was performed following standard protocol as previously described [26]. CD29, CD44, Sca-1 and CD90, CD45, CD34 CD4 antigens (all antibodies from BD Biosciences, conjugated to FITC or PE) were evaluated. Intracellular staining was also performed to determine the expression of IL6, iNOS, and COX-2 (all antibodies from BD Biosciences). Previously, the cells were fixed and permeabilized (Foxp3/Transcription Factor Staining Buffer Set from eBioscience, USA). Moreover, for the T helper differentiation analysis, cell viability and surface staining were assessed using LIVE/DEAD dye (Invitrogen, USA). Cells were stained with anti-IFNγ-FITC for Th1, anti-IL17A-PE for the Th17 and Tregs with anti-CD25-FITC plus anti-Foxp3-PE. T cells were acquired with a FACSCanto II cytometer and analyzed using the Flow Jow Star software.

### Immunosuppression assay

Splenocytes were isolated from the C57BL/6 mouse spleen and stained with CellTrace Violet (CTV) following the manufacturer’s instructions. CTV-splenocytes were stimulated with concanavalin A (1 μg/ml) and co-cultured with MSCs in a 1:10 ratio (MSCs to splenocytes) in RPMI medium containing 10% fetal bovine serum (FBS) (Lonza, Maryland, USA), 2 mM l-glutamine, 50 μM β-mercaptoethanol, 100 U/mL penicillin, and 100 μg/mL streptomycin (Gibco, Paisley, UK) at 37 °C in a 5% CO_2_ atmosphere. T cell proliferation was assayed on viable (CTV negative) CD4+IFNγ, CD4+IL17+, and CD4+CD25+Foxp3+ T cells by flow cytometry.

### Treg, Th17, and Th1 differentiation in vitro

Purified CD4+T cells using a CD4+ T cell Isolation Kit (Miltenyi Biotec, Bisley, UK) according to the manufacturer’s instructions were cultured in RPMI medium supplemented with 10% heat-inactivated FBS, 2 Mm l-glutamine, 1 mM sodium pyruvate, 20 mM HEPES, and 50 μM of β-mercaptoethanol (Invitrogen, Grand Island, NY, USA), 100 U/ml penicillin, and 100 μg/ml streptomycin (GIBCO) at 37 °C in a 5% CO2 incubator and activated with antibodies anti-CD3/CD28 (1 μg/ml, each) (BD Pharmigen, USA). Treg differentiation was added 5 ng/mL TGF-b (Peprotech, Germany) and 2 ng/mL IL2 (eBiosciences, USA). Th17 cells differentiation were induced with 2.5 ng/ml TGFβ1 (Peprotech, Germany), 20 ng/ml IL6 (R&D System, USA), and 2.5 μg/ml of both anti-IFNγ and anti-IL4 capture antibodies (BD Biosciences). Th1 differentiation was induced by adding 5 ng/ml of IL12 and 2.5 μg/ml of anti-IL4 (BD Biosciences). After 3 days of culture or co-culture with MSC, cell proliferation was measured by flow cytometry. T cells were stimulated for 4 h with 50 ng/ml phorbolmyristate acetate (PMA) (Sigma-Aldrich), 1 μg/mL Ionomycin (Sigma-Aldrich), and 10 μg/mL Brefeldin A (Sigma-Aldrich). CD4-Percp5.5, IFNγ-FITC, IL17-PE, CD25-APC, and FOXP3-PE antibodies were used (all from BD Biosciences).

### EAE protocol

Ten- to 14-week-old female C57BL/6 mice were used to induce EAE in vivo model, and MSCs were subcutaneously (s.c.) injected. In previous studies, we characterize this procedure which is detailed in Additional file 1: Appendix A. Animal weight and clinical signs of disease were evaluated daily. Clinical and cumulative scores were calculated as previously described [13] and are showed in Additional file 1: Appendix A.

### T helper analysis in lymph nodes of EAE mice

At the end of the EAE protocol, inguinal, axillary, and neck lymph nodes were harvested for immunological analysis. Cell suspension was cultured overnight (3 × 10^6^ cells/ml) in complete RPMI medium and stimulated with PMA/Ionomycin (50 ng/ml and 1 μg/ml, respectively) for 3 h and Brefeldin A. Lymphocytes analysis) CD4+IFNγ, CD4+IL17+, and CD4+CD25+Foxp3+ T cells by flow cytometry were performed. Violet live-dead dye (Invitrogen), CD4-Percp-Cy5.5, IFNγ-FITC, IL17-PE, CD25-FITC, and FOXP3-PE antibodies were used (all from BD Biosciences).

### Statistical analyses

Statistical analyses were performed using GraphPad Prism 5.0 software (San Diego, CA, USA). Data were expressed as mean ± SEM. One way-ANOVA was used to compare differences of data from more than two groups. For non-parametric data, the Kruskal-Wallis test was used. EAE scores were analyzed by two way-ANOVA. All *p* values < 0.05 were considered statistically significant.

## Results

### Time-dependent LPS activation regulate IL6 and iNOS expression in MSCs

It has been previously described that iNOS (enzyme responsible for secreting nitric oxide, NO) is a key molecule in mouse MSCs exerting the immunosuppressive effects. Conversely, IL6 secreted by MSCs has been associated with a decrease in their immunosuppressive potential. Immunosuppressive MSCs (MSC2) are associated with an increased expression of anti-inflammatory molecules and are capable of inhibiting the proliferation of effector T lymphocytes and also inducing regulatory T lymphocytes, FOXP3 +. Pro-inflammatory MSCs (MSC1) induce rather pro-inflammatory molecules and activate lymphocyte proliferation, preventing the induction and activation of regulatory T lymphocytes [22, 26, 28, 29].

**FIG. 1:**
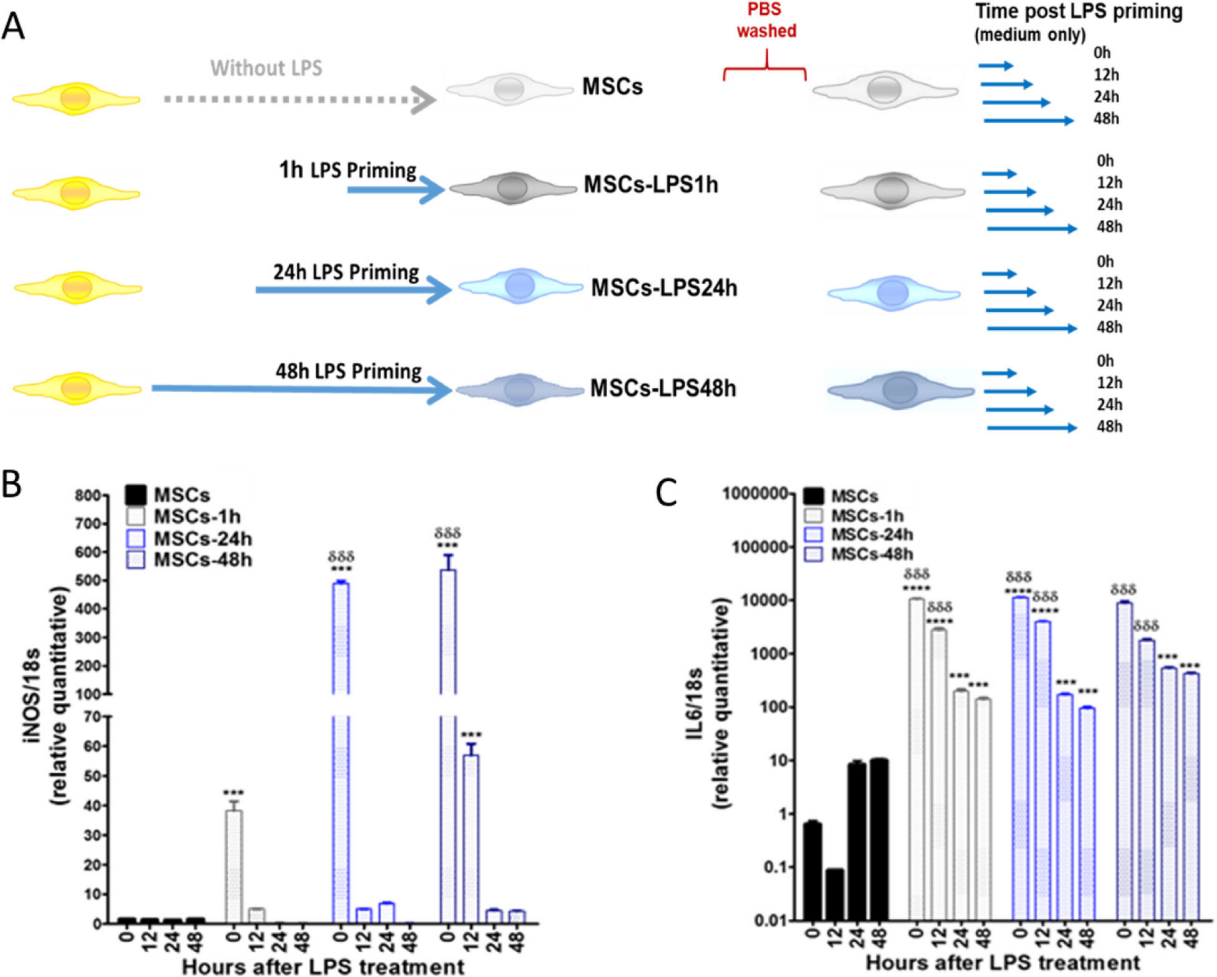
Long LPS stimulation in MSCs increases the expression of inducible nitric oxide synthase (iNOS). **a** MSCs were stimulated with LPS (500 ng/ml) for 1, 24, and 48 hours (h), then washed with PBS and cultured again in complete α-MEM medium for next 12, 24, and 48 h. **b** RNA was obtained, and quantitative PCR (qPCR) assays for iNOS and **c** IL6 genes were performed. Data are expressed as mean ± SEM; *n* = 3, *N* = 3 biological replicates; ***p < 0.001, ****p < 0.0001 (inside each experimental group). δδδ*p* < 0.001 (between each experimental group), derived by one-way ANOVA, Kruskal-Wallis ad hoc post test

It has been demonstrated that MSCs in a pro-inflammatory environments, such as INFγ; IL6 expression induces PGE2 secretion promoting an anti-inflammatory phenotype of MSCs [30]. However, when MSCs are stimulated with LPS for 1 h, higher expression of IL6 is translated into a pro-inflammatory phenotype of MSCs (MSC1) [22]. Therefore, we assessed whether differential LPS stimulation under short (1 h) or long exposure (24 and 48 h) induces a change in the expression levels of iNOS and IL6 (schematic representation of the experimental design is showed in Fig. 1a). Our results showed a 500-fold increase in iNOS expression in MSCs by both MSCs exposed to LPS for 24 h (MSCS-LPS24h) (1.4 ± 0.05 vs. 488.8 ± 9.4) and 48 h (MSCs-LPS48h) (1.4 ± 0.1 vs. 536.4 ± 52.6) but not in MSCs exposed to LPS for 1 h (MSCs-LPS1h) (1.4 ± 0.1 vs. 38.3 ± 3.2) (***p < 0.001) (Fig. 1b). Interestingly, when LPS stimulation was removed, we observed a rapid decrease of iNOS expression reaching basal levels in MSCs-LPS24h (1.4 ± 0.1 vs. 56.9 ± 4.0) (*** *p* < 0.001). Additionally, IL6 expression showed a higher 10.000-fold increase in MSCs-LPS1h (0.6 ± 0.1 vs. 10,500.5 ± 307.9) and MSCs-LPS24h (0.6 ± 0.1 vs. 11,368.9 ± 246.4), compared to the basal condition (****p < 0.0001). Nevertheless, IL6 expression in MSCs-LPS48h increases to a lesser degree (0.6 ± 0.1 vs. 8915.6 ± 867.4) (***p < 0.001) (Fig. 1c). Of note, TLR4 expression levels were increased upon LPS activation independent of exposure time (Supp. Fig. 3).

### The immunosuppressive activity of MSCs on T cell proliferation depends on LPS activation in a time-dependent manner

**Fig. 2:**
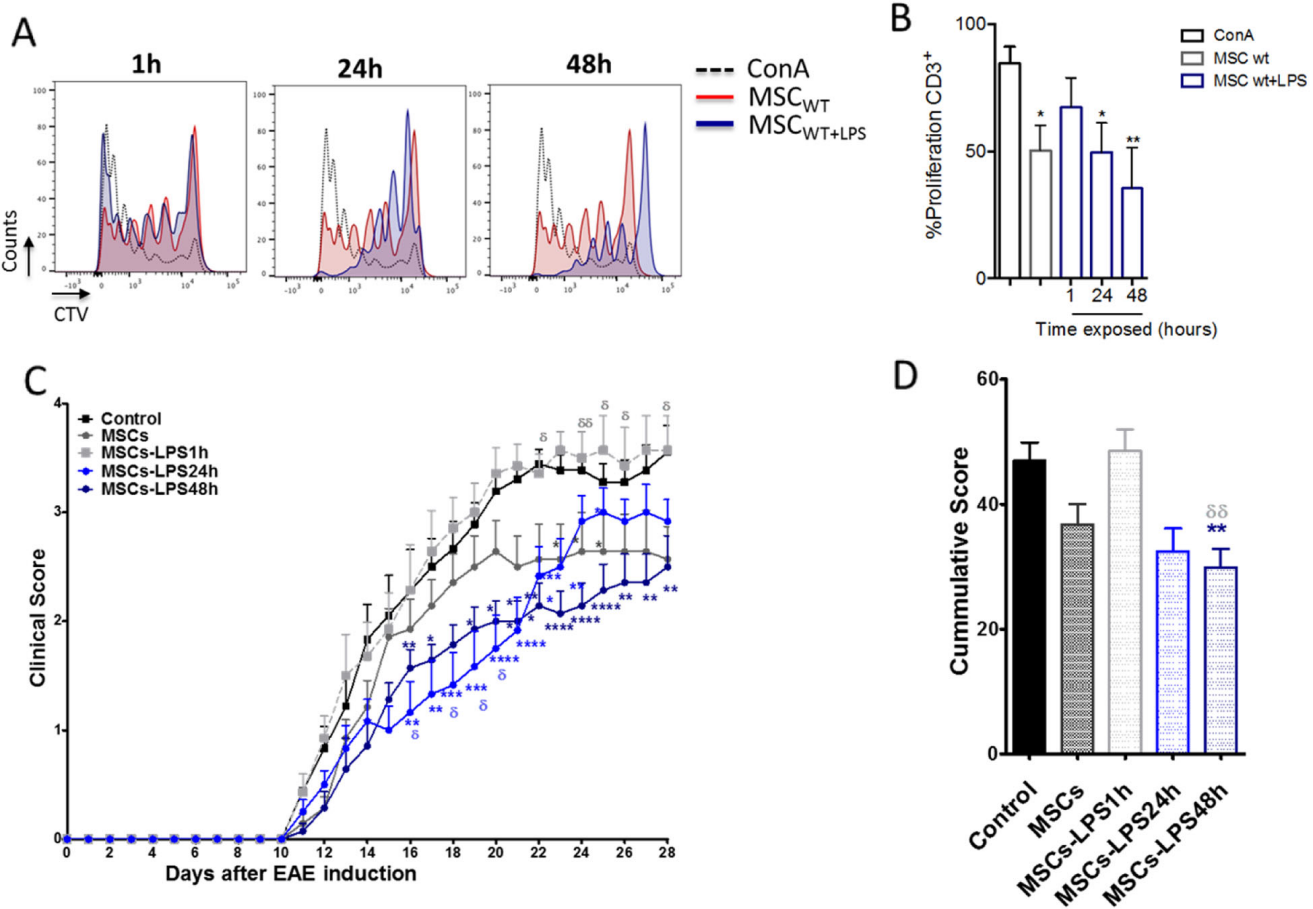
Long LPS exposure significantly increases the therapeutic efficacy of MSC. MSCs were priming in the presence or absence of LPS for 1, 24, and 48 h and coculture with splenocytes that were previously stained with the fluorescent dye CellTrace Violet (CTV) and activated for 72 h with concanavalin A (ConA, 1 μg/ml). **a** Representative histogram analysis of T cell proliferation by flow cytometry. **b** Percentage of CD3+ T cells proliferation (%) after coculture with or without MSCs. Data are expressed as mean ± SEM; *n* = 3, *N* = 3 biological replicates; *p < 0.05, **p < 0.01. Statistical analysis was performed by one-way ANOVA, Kruskal-Wallis ad hoc post test. **c**, **d** EAE in vivo model: MSCs after priming with LPS (500 ng/m for 1 and 24 or 48 h) were injected 7 days after EAE induction. Clinical symptoms and weight loss were evaluated daily. **c** Clinical score and **d** cumulative clinical score are shown. Representative data of three independent experiments. Data are expressed as mean ± SEM; *n* = 12. * symbol represent the comparison between EAE control group and those groups treated with MSCs (*p < 0.05, **p < 0.01, ***p < 0.001, ****p < 0.0001). δ symbol represent the comparison of EAE+MSCs group versus LPS-treated MSCs groups. (δδ < 0.01). Statistical analysis was performed by **c** two-way ANOVA or **d** one-way ANOVA, Kruskal-Wallis ad hoc post test

In order to evaluate the effect of LPS activation on MSCs’ suppressive function, we performed T cell proliferation assay in vitro. For that purpose, splenocytes stained with CellTrace Violet (CTV) and activated with Concanavalin A were used to evaluate T cell proliferation. T cells were co-cultured for 3 days with MSCs stimulated with LPS at different time points (1 h, 24 h, and 48 h). Our results showed that T cell proliferation was significantly impaired when MSCs were exposed to LPS for 1 h. In contrast, when MSCs were pretreated with LPS for 48 h, they improve their suppressive activity compared to non-treated MSCs (Fig. 2a, b). Moreover, NO secretion was determined in the supernatants of co-cultures of MSCs:T, and we observed a lower NO secretion in MSCs-LPS1h compared to both control MSCs and MSCs-LPS48h (***p < 0.001) (Supp. Fig. 4).

### Long exposure to LPS enhances MSCs’ therapeutic potential in EAE and is overturned by short exposure

Next, we sought to investigate the therapeutic potential of MSCs differentially stimulated with LPS using the in vivo EAE model. Our results showed that while MSCs-LPS1h display no effect on the clinical score of EAE animals compared to untreated MSCs. MSCs-LPS48h significantly improve the clinical score of EAE mice compared to untreated MSCs (Fig. 2c). MSCs-LPS1h have a higher cumulative score versus EAE non-treated (29.9 ± 3.0 vs. 46.9 ± 3.0, days/animal, **p < 0.01). Cumulative score of MSCs-LPS48h was lower than EAE treated with unstimulated MSCs (29.9 ± 3.0 vs. 21.9 ± 3.9 days/animal, δδ *p* < 0.01) (Fig. 2d). Of note, even that MSCs-LPS24h showed a better protective effect during the first 10 days of the onset of the diseases, this effect was subsequently lost (Fig. 2c). Contrary to MSCs-LPS48h, that maintains the clinical effect over time. The weight of the animals was evaluated daily, showing no significant differences (Supp. Fig. 5).

### TLR4 expression is involved in the immunosuppressive capacity of MSCs in vitro

Based on the obtained results suggesting that LPS could induce either pro- or anti-inflammatory MSC phenotype depending on the exposure time, we evaluated the contribution of TLR4 on MSCs’ biology and suppressive function. MSCs deficient for TLR4 (MSCs-TLR4KO) were obtained from B6.B10ScN-Tlr4lps-del/JthJ mice and MSCs WT from their corresponding littermate wild type. Phenotypic characterization, differentiation potential, and genotypic analysis for both MSCs were performed (Supp. Fig. 2).

**Fig. 3:**
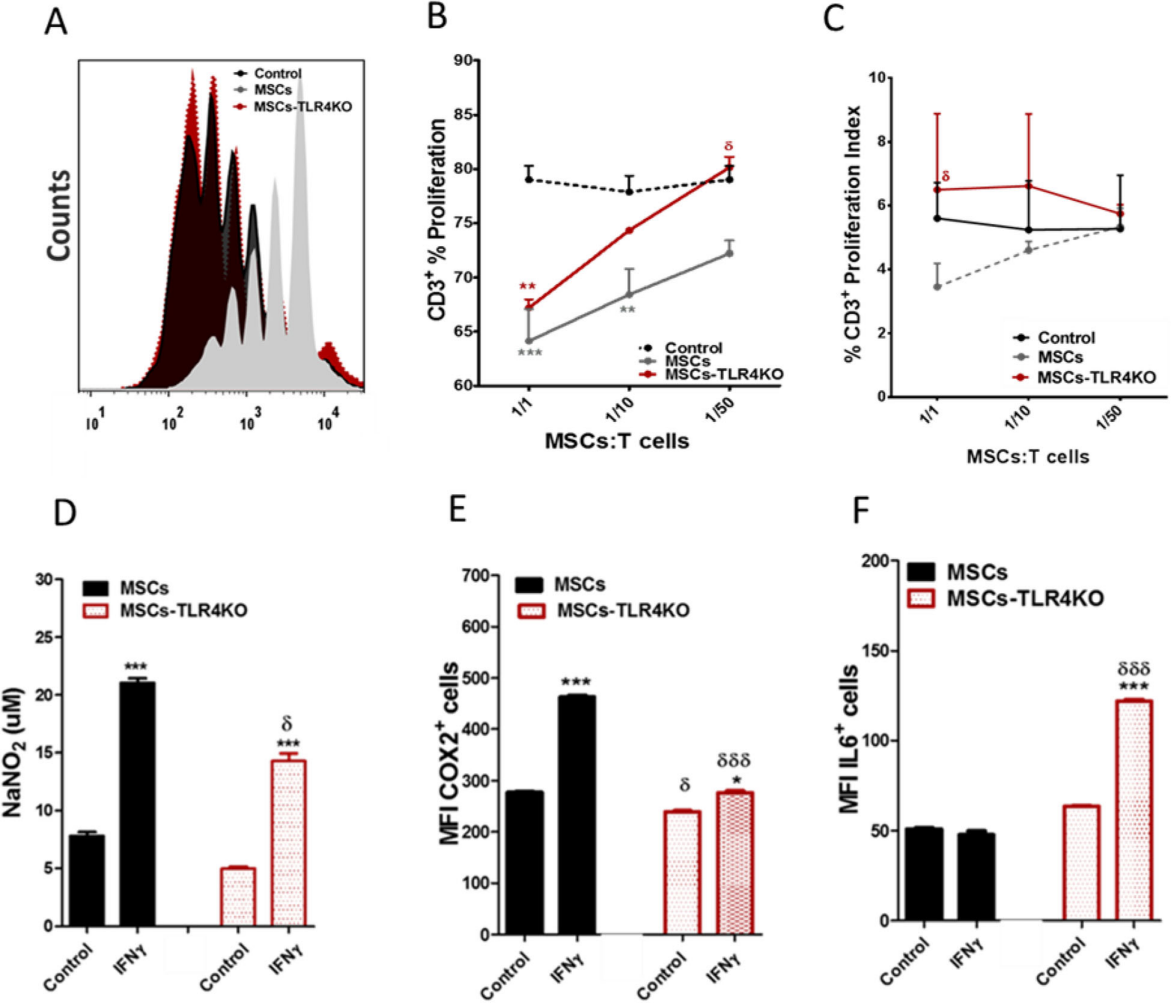
TLR4 expression mediates the immunosuppressive capacity of MSCs in vitro. CTV labeled splenocytes were cultured alone or with either MSCs WT or MSCs-TLR4KO at different MSCs: T cell ratio (1/1, 1/10, and 1/50) and activated with 1 μg/ml of ConA for 72 h. T cell proliferation was evaluated by FACS analysis. **a** Representative histograms for T cell proliferation with or without MSCs WT or MSCs-TLR4KO. Proliferation was calculated according to **b** frequency of total CD3+ T cells proliferation or **c** proliferation Index. **d** NO production was detected in the supernatants of MSCs WT or MSCs-TLR4KO using a modified Griess reagent. **e** COX2 and **f** IL6 expression was analyzed by flow cytometry in MSCs WT or MSCs-TLR4KO, pretreated or not with IFNγ for 48h. Data are expressed as mean ± SEM; *n* = 3, *N* = 3 biological replicates; *p < 0.05, ***p < 0.001 (inside each experimental group). δ*p* < 0.05, δδδ*p* < 0.001 (between each experimental group), derived by one-way ANOVA, Kruskal-Wallis ad-hoc post test

The loss of the expression of TRL4 (MSCs-TLR4KO) mitigates the MSC capacity to inhibit T cell proliferation, compared to wild-type MSCs, both in their CD3+ T cell proliferation frequency and index proliferation (Fig. 3a–c). This was associated with a significantly lower capacity to express suppressive mediators such as iNOS (Fig. 3d) and cyclooxygenase 2 (COX2) (Fig. 3e) upon IFNγ activation (iNOS: MSCs vs. MSCs+IFNγ: 201.1 ± 0.5 vs. 340.78 ± 8.4 and MSCs-TLR4KO vs. MSCs-TR4KO+IFNγ: 187.1 ± 1.0 vs. 263.3 ± 4.1; *p < 0.05; and COX2: MSCs vs. MSCs+IFNγ: 277.0 ± 1.8 vs. 462.0 ± 2. 5 and MSCs-TLR4KO vs. MSCs-TR4KO+IFNγ: 238.5 ± 1.9 vs. 275.8 ± 3.5; *p < 0.05 and ***p < 0.001). Moreover, we also observed that IFNγ-primed MSCs-TLR4KO showed a substantial increase of IL6 expression (IL6: MSCs+IFNγ vs. MSCs-TR4KO+IFNγ: 47.9 ± 2.0 vs. 121.8 ± 1.0 MFI, δδδ*p* < 0.001) compared to wild-type MSCs (Fig. 3f). Also, no change in NO production was observed when MSCs-TLR4KO were activated with LPS for 24 h (Supp. Fig. 6). These results associate the KO of TLR4 expression with the loss of inhibition of T cell proliferation. Furthermore, MSCs-TLR4KO display lower expression levels of iNOS and COX2 and higher expression of IL6 in comparison with wild-type MSCs.

### TLR4 inhibition disrupts the capacity of MSCs to inhibit Th1 and Th17 cells in vitro

**Fig. 4:**
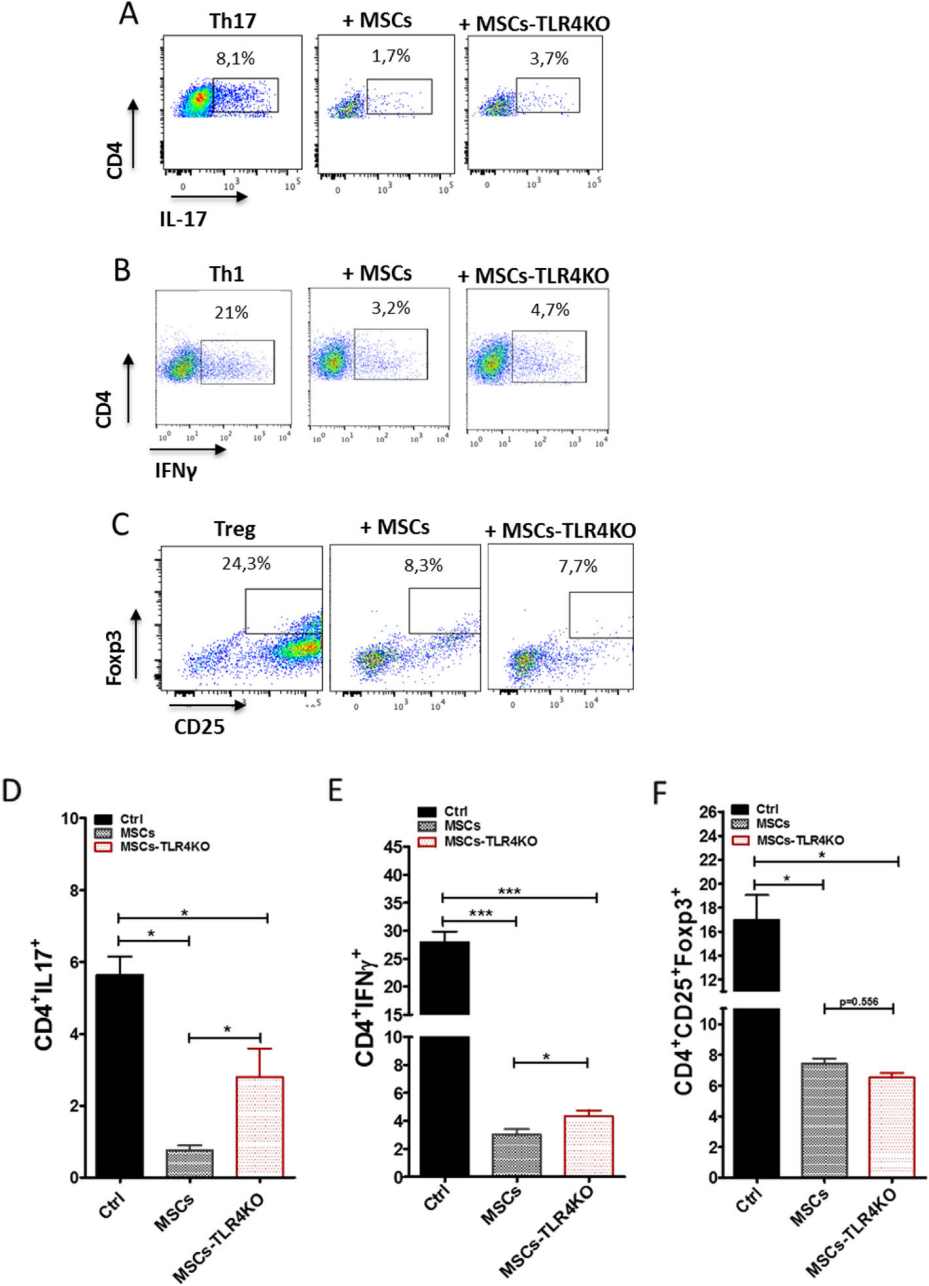
MSCs-TLR4KO has a limited capacity to inhibit pro-inflammatory Th1 and Th17 cells in vitro. Freshly isolated Naïve T-CD4 cells were differentiated into Th17, Th1, and Treg cells and co-cultured or not with MSCs WT or MSCs-TLR4KO. **a** Representative dot plot of IL17+, **b** IFNγ+ and **c** CD25+Foxp3+ cells on T CD4 cells prompted to in vitro differentiation. Quantification of **d** IL17- and **e** IFNγ-producing cells by FACS analysis. **f** Quantification of CD25+Foxp3+ positive cells. *p < 0.05, **p < 0.01, ***p < 0.001 (control vs. MSCs). Statistical analysis was performed by one-way ANOVA, Kruskal-Wallis ad hoc post test

In order to evaluate the effect of TLR4 expression on pro-inflammatory T cells, we perform co-culture experiments using MSCs and MSCs-TLR4KO with freshly isolated CD4 T cells induced to differentiate into Th1, Th17, and Tregs. For that purpose, after 3 days of co-culture, Th1 (CD4+IFNγ+), Th17 (CD4+IL17+), and Treg (CD4+CD25+Foxp3+) generated in vitro were evaluated by FACS analysis. The in vitro results showed that MSCs-TLR4KO partially reverse the immunosuppressive capacity to inhibit the differentiation towards Th17 and Th1 lymphocytes compared to MSCs WT (Th17: 0.8 ± 0.1 vs. 2.9 ± 0.3, *p < 0.05 (Fig. 4a, d), and Th1: 2.1 ± 0.2 vs. 5.0 ± 0.4, *p < 0.05 (Fig. 4b, e)). In addition, MSCs-TLR4KO suppresses the generation of Tregs at the same levels than wild-type MSCs (7.5 ± 0.4 vs. 7.2 ± 0.7) (Fig. 4c, f). In vitro, MSCs and MSCs-TLR4KO inhibit both Th17 and Th1 pro-inflammatory lymphocytes; however, significant differences are found between two different types of MSCs (*p < 0.05).

Taken together, the results obtained suggest that MSCs-TLR4KO partially lost their immunosuppressive properties in vitro, hence suggesting a MSC1-like phenotype in comparison to MSCs WT.

### TLR4 deficiency forfeits the therapeutic effect of MSCs in EAE

**Fig. 5:**
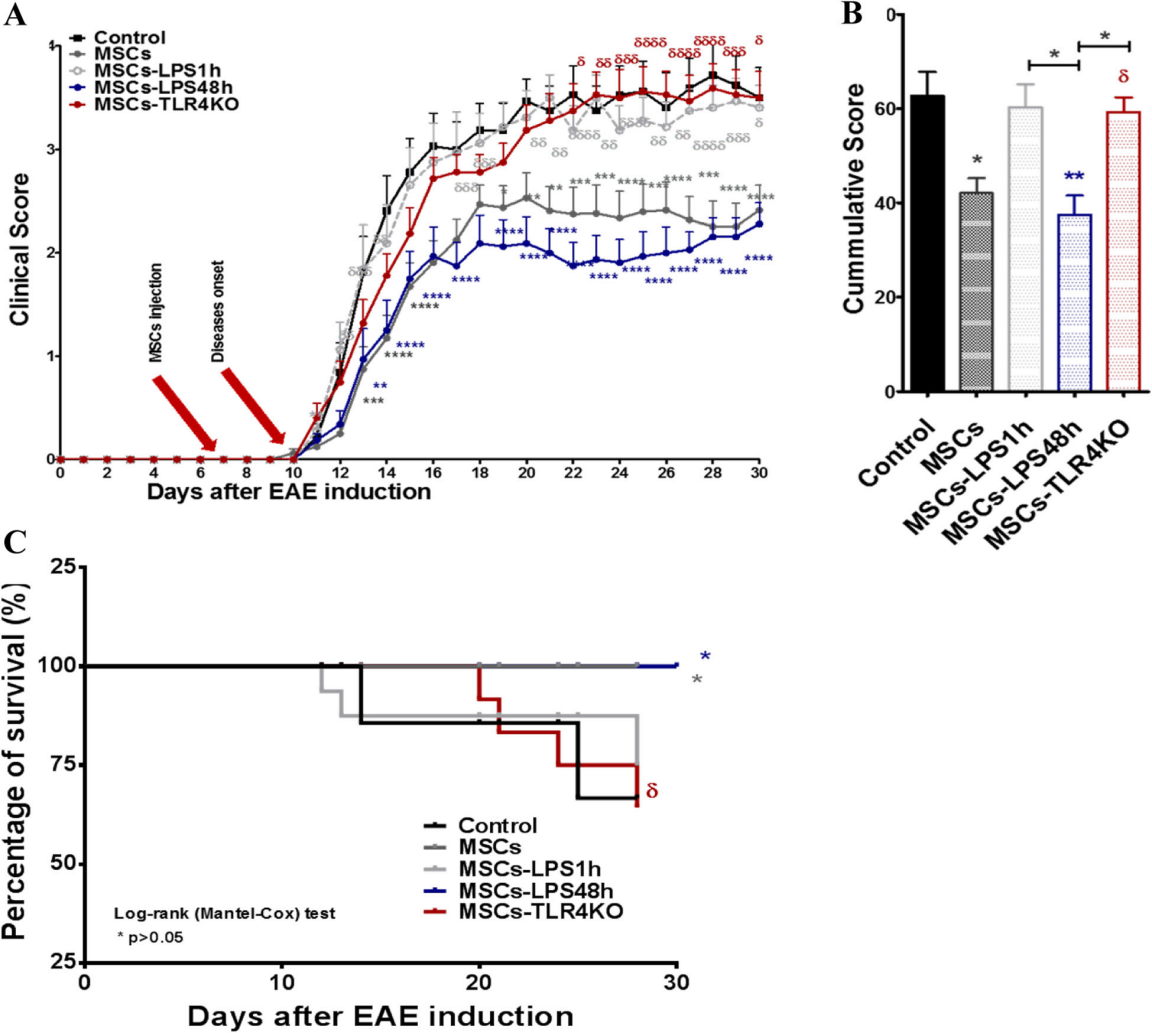
MSCs-TLR4KO loss their therapeutic potential in EAE. MSCs-TLR4KO or MSC WT primed or not with LPS (500 ng/m for 1 or 48 h) were injected 7 days after EAE induction. Clinical symptoms and weight loss were evaluated daily. **a** Clinical score, **b** cumulative clinical score, and **c** Kaplan-Meier curve for survival evaluation. Representative data of three independent experiments. Data are expressed as mean ± SEM; *n* = 12. * symbol represents the comparison against EAE control group. *p < 0.05, **p < 0.01, ***p < 0.001, ****p < 0.0001. δ symbol represents the comparison EAE+MSCs group vs. MSCs treated with LPS. δ *p* < 0.05, δδ *p* < 0.01, δδδ *p* < 0.001, δδδδ *p* < 0.0001. Statistics used was two-way ANOVA

To better understand the role of TLR4, in vivo experiment using the EAE murine model was performed considering the following experimental groups: Control group (EAE without MSCs), unstimulated MSCs, MSCs-LPS1h, MSCs-LPS48h, and MSCs-TLR4KO. Consistent with our previous results, MSCs induced a significant improvement in the daily clinical score contrary to MSCs-LPS1h and MSCs-TLR4KO that overturned their therapeutic effect (*p < 0.05, **p < 0.01, ***p < 0.001, ****p < 0.0001) (Fig. 5a). Cumulative clinical score analysis showed EAE, MSCs-LPS1h, and MSCs-TLR4KO have the highest clinical score (48.5 ± 3.7, 46.7 ± 3.1 and 47.4 ± 2.9, respectively) compared to control MSCs and MSCs-LPS48h (33.9 ± 3.3 and 29.1 ± 3.0, respectively) (*p < 0.05, δ*p* < 0.05) (Fig. 5b). Indeed, MSCs-LPS48h decrease the clinical score more significantly than MSCs. In line with these observations, the percentage of survival, as was shown in Kaplan-Meier Curve, was also affected, where MSCs and MSCs-LPS48h showed 100% survival rates compared to untreated animals (75% survival) (*p < 0.05). On the other hand, MSCs-TLR4KO showed a faster decrease in animal survival rate than animals treated with control MSCs (δ*p* < 0.05) (Fig. 5c). These results confirm that MSCs-TLR4KO display a MSC1 phenotype similar to MSCs-LPS1h. On the other hand, long LPS stimulation (MSCs-LPS48h) led to an anti-inflammatory response suggesting a MSC2-like phenotype. The weight of the animals was evaluated daily, showing no significant differences (Supp. Fig. 5). Moreover, MSCs-TLR4KO treated with LPS for 1 and 24 h showed no differences compared to untreated MSCs-TLR4KO, and all of them showed a similar increase in the clinical score of the EAE animals (Supp. Fig. 7).

**Fig. 6:**
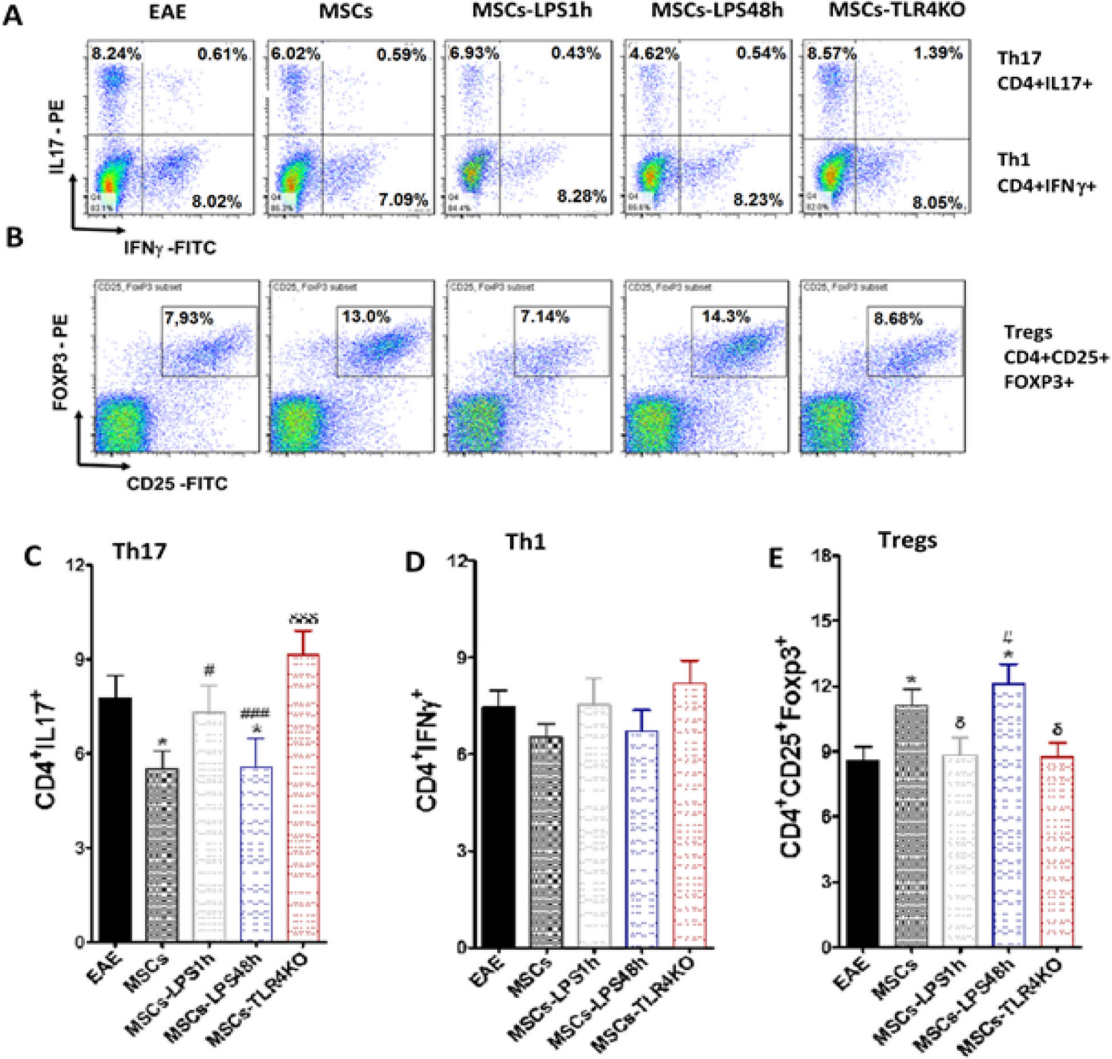
Short LPS activation and TLR4 deletion impair the immunosuppressive capacity of MSCs in EAE mice. EAE mice were treated or not with MSCs WT, MSCs-TLR4KO, or MSCs pretreated with LPS for 1 h or 48 h. T lymphocytes derived from lymph nodes of EAE mice were analyzed by FACS at euthanasia. Representative dot plot of **a** Th17: CD4+IL17+, and Th1: CD4+IFNγ+ and **b** Tregs: CD4+CD25+FOXP3+ were analyzed. **c** Th17, **d** Th1, and **e** Treg total frequency were evaluated. *p < 0.05 (EAE control vs. LPS treated MSCs or MSCs-TLR4KO). δ *p* < 0.05, δδδ *p* < 0.001 (MSCs vs. LPS-treated MSCs or MSCs-TLR4KO). ^#^*p* < 0.05 (LPS-treated MSCs vs. MSCs-TLR4KO). Statistics use was one-way ANOVA Kruskal-Wallis ad hoc post test

Finally, we analyze the T lymphocyte subpopulations in the lymph nodes of the EAE animals treated with the different types of MSCs at the time of euthanasia. Flow cytometer analysis was performed to Th1, Th17, and Treg lymphocytes (Gating strategy in Supp. Fig. 8B). We observed a decreased percentage of Th17 in mice injected with MSCs (7.8 ± 0.7 vs. 5.5 ± 0.6) (*p < 0.05) and MSCs-LPS48h (7.8 ± 0.7 vs. 5.6 ± 0.9) (*p < 0.05) compared to untreated animals. On the other hand, an increased frequency of pro-inflammatory Th17 lymphocytes was detected in the lymph nodes of mice treated with MSCs-TLR4KO compared to mice treated with MSCs (9.7 ± 0.8 vs. 5.5 ± 0.6) (δδδ *p* < 0.001). Additionally, MSCs-TLR4KO showed the highest percentage of Th17 lymphocytes compared to both MSCs-LPS1h and MSCs-LPS48h (6.8 ± 0.8 vs. 9.7 ± 0.8, # *p* < 0.05, and 5.6 ± 0.9 vs. 9.7 ± 0.8, ### *p* < 0.001, respectively) (Fig. 6a, c). This indicates that highest pro-inflammatory phenotype is attained under condition where TLR4 expression is entirely abrogated. We did not found any significant difference in Th1 lymphocyte (Fig. 6a, d). In contrast, we observed a significant increase in Treg in animals treated with MSCs and MSCs-LPS48h compared to untreated animals (8.5 ± 0.6 vs. 12.2 ± 1.0, *p < 0.05; 8.5 ± 0.6 vs. 12.3 ± 0.9, *p < 0.05, respectively). Further, we observed a significant decrease of Treg lymphocytes in MSCs-LPS1h and MSCs-TLR4KO compared to MSC-treated mice (9.0 ± 0.8 vs. 12.2 ± 1.0, δ*p* < 0.05, and 8.3 ± 0.5 vs. 12.2 ± 1.0, δ*p* < 0.05, respectively). Finally, we observed highest levels of Treg lymphocytes in MSCs-LPS48h compared to MSCs-TLR4KO (12.3 ± 0.9 vs. 8.3 ± 0.5) (#*p* < 0.05) (Fig. 6b, e). The aforementioned result is correlated with a pro-inflammatory response induced by the MSCs-LPS1h and MSCs-TLR4KO in the treated animals and the worsening of the EAE symptoms, in contrast with MSCs and MSCs-LPS48h.

In conclusion, MSCs and MSCs-LPS48h inhibit Th17 compared to untreated animals (*p < 0.05); only MSCs-LPS48h, but not MSCs, inhibit more efficiently Th17 compared to MSCs-TLR4KO (###*p* < 0.001); MSCs-LPS1h fail to retain the ability to inhibit Th17 in comparison to MSCs-TLR4KO (#*p* < 0.05); and MSCs-TLR4KO induces Th17 compared to wild-type MSCs (δδδ*p* < 0.001). No differences were found on Th1 lymphocytes. Also, MSCs and MSCs-LPS48h induce Treg (*p < 0.05). MSCs-LPS48h induce higher Treg infiltration in the lymph nodes of EAE animals compared to MSCs-TLR4KO (#*p* < 0.05), contrary to MSCs-LPS41h and MSCs-TLR4KO which are enable to induce Treg compared to MSCs (δ*p* < 0.05). In contrast with MSCs-TLR4KO, MSCs and MSCs-LPS48h inhibit Th17 induction in EAE and increase Tregs.

## Discussion

While TLR4 activation has been demonstrated to play a fundamental role in the induction of pro-inflammatory phenotypes in DCs and Macrophages [23–25], our results show its relevance in MSCs’ biology and function. In this study, we reveal that MSCs can acquire differential pro- or anti-inflammatory phenotypes in a time-exposure-dependent manner. Moreover, we showed that TLR4 expression in MSCs is critical for MSCs to maintain their immunosuppressive properties. MSCs-LPS48h showed a higher immunosuppressive potential through the expression of higher levels of NO and inhibition of T cell proliferation leading to an enhanced therapeutic effect in EAE, inducing an anti-inflammatory response with higher expression of Treg in the lymph nodes of EAE animals. Our results are in line with previous observation were TLR4 activation of MSCs was associated to Treg induction via the Notch signaling in inflammatory environments [29, 31]. On the other hand, MSCs-LPS1h showed a lower NO production, lost the capacity to inhibit T cell proliferation in vitro leading to a significant loss of the therapeutic effect in EAE. This preclinical outcome shows that a phenotype conversion following the short LPS exposure as the anti-inflammatory properties are lost over the course of the experiment. MSCs-LPS48h secrete the highest NO levels, inhibit T cell proliferation more efficiently, and have the greatest therapeutic potential in the EAE model. These results suggest that MSCs-LPS48h have a MSC2-like phenotype. By contrast, MSCs-LPS1h secrete lower amounts of NO and fail to retain the ability to inhibit T cell proliferation in vitro, resulting in a loss of their therapeutical effect in EAE model, which suggests a MSC1 phenotype.

Thus, in order to understand the mechanism behind the different phenotype changes, MSCs-TLR4KO were used. Our results showed that MSCs-TLR4KO display a higher capacity to produce IL6 together with a lower expression of immunosuppressive mediators like iNOS and COX2 and a lower capacity to inhibit T cell proliferation compared to control MSCs. In addition, it is observed that MSCs and MSCs-TLR4KO inhibit both Th17 and Th1 pro-inflammatory lymphocytes; however, significant differences are found between two different types of MSCs. All these immunophenotypic changes compromised their therapeutic effect in EAE and a decreased in animal survival rate, which were correlated with an increase of Th17 and reduction of Treg lymphocytes in vivo, hence displaying a MSC1-like phenotype. The molecular mechanism on how exactly TLR4 respond to same pro-inflammatory stimuli at different exposure times remains to be deciphered. MSCs and MSCs-LPS48h inhibit Th17 and induce Treg in vivo compared to untreated animals. However, MSCs-LPS48h prevents Th17 and induces higher Treg infiltration to the lymph nodes in EAE compared to MSCs-TLR4KO. We noted differences between the in vitro and in vivo results regarding the effect of MSCs or MSCs-TLR4KO on Treg lymphocytes. Although no important difference was detected between MSCs and MSCs-TLR4KO in vitro, the in vivo results were considered more reliable in reflecting the inflammatory microenvironment and for assessing the immunosuppressive capacity of MSCs with respect to Treg differentiation.

TLR pathways are tightly regulated by multiple mechanisms. Activation of TLR4 requires a cascade of events starting from an interaction of LPS with series of adaptors and co-receptors (LBP, MD2, and CD14) to allow dimerization of TLR4 through TIR domains. These facilitate recruitment of two pairs of adaptor proteins, TIRAP/MyD88 (MyD88-dependent pathway) and/or TRAM/TRIF (MyD88-independent pathway). MyD88-dependent pathway classically induces a cascade of events to involve activation of nuclear factor kB (NF-kB), Janus kinase-phosphoinositide 3-kinase (JAK-PI3K), and mitogen-activated protein kinase (MAPKs) which could finally activate activator protein 1 (AP1) signals, and regulate the production of pro-inflammatory cytokines like TNF, IL6, iNOS, etc. Separately, MyD88-independent pathway can also initiate a signaling pathway which directly activates interferon regulatory factor 3 (IRF3) transcription factor, leading to the expression of type I interferon’s (IFNs). MyD88-dependent pathway has also included the activation of other transcription factors associated to macrophages plasticity very well described by Lawrence and Natoli [32]. However, to date, those signaling pathways have not been corroborated in MSCs yet. In macrophages, the expression of cAMP-responsive element-binding protein (CREB) [33] inhibits AP1, which are involved in the expression of iNOS, IL6, TNF, and IL1 [34]. Additionally, CREB expression has been described in MSCs, regulating COX2 function [35].

Moreover, we cannot conclude that the MSC2 phenotype induced by long exposure to LPS is mediated only by the TLR4 signaling pathway. Indeed TLR4 can activate both signaling pathways (dependent or independent to Myd88) [36]. In this case, the absence of TLR4 may be compensated with the Myd88-independent pathway, in which activation occurs through a domain called TRIF and TRAF3, leading to the recruitment of IKKΣ and TBK-1 then IRF3 activation by phosphorylation and consequently the release of anti-inflammatory cytokines which induces IFN-β expression, essential for this pathway [36].

In this context, the MSCs-TLR4KO lose the possibility of being activated with LPS through the Myd88-dependent/TRAF6/NFκb activation pathway, preventing prior priming with this molecule and then preventing the proper licensing of the MSCs with pro-inflammatory cytokines in vitro and in vivo under a pro-inflammatory environment. The results obtained from the EAE model suggest that the absence of TLR4 in MSCs induces a more robust MSC1 phenotype in comparison with the treatment of MSCs with 1-h LPS. Future studies focusing on the downstream activated molecular pathway can confirm whether the MSC2 phenotype induced by long exposure to LPS is fully or partially dependent of the TLR4 pathway.

Micro-RNAs (miRNAs) are also critical factors in the regulation of TLR4 response, mostly by inhibiting the NF-kB pathway. Previously, it has been described that MSC1 have a high expression of miR-155, contrary to MSC2 that maintains low levels [37]. Interestingly, it is mentioned that miR-155 negatively regulates iNOS production in MSCs by targeting TAB2 mRNA [38], which forms a complex with TAB3 and TAK1 that allows activation of NF-kB and miR-155 blocking TAB2 inhibit NF-kB activation [37]. Waterman et al. showed that high levels of miR-155 are expressed in MSC1 (MSCs-LPS1h). It is well known that one of the major transcription factors that induce the expression of iNOS is mediated by NF-kB [39]. miR146a has been described in monocytes to be responsible of targeted IRAK1and TRAF6, suppressing also TLR4 signaling pathway [40]. Moreover, it is mentioned that TLR4 activation increase also the expression of miR-Let7b [41], but this was associated with an induction of type 2 macrophages via extracellular vesicle delivery [41]. We propose miR-Let7b could also be involved in MSCs’ immunoplasticity. On the other hand, few miRNAs are upregulated downstream TLR4 activation. Increased expression of miR-301a in MSCs was associated with an upregulation of IL8, COX2, PGE2, IFNβ, and IDO [42], master regulators of the immune-modulatory properties of MSCs. Abdi et al. well-reviewed the roll of miRNAs in MSCs [43].

**Fig. 7:**
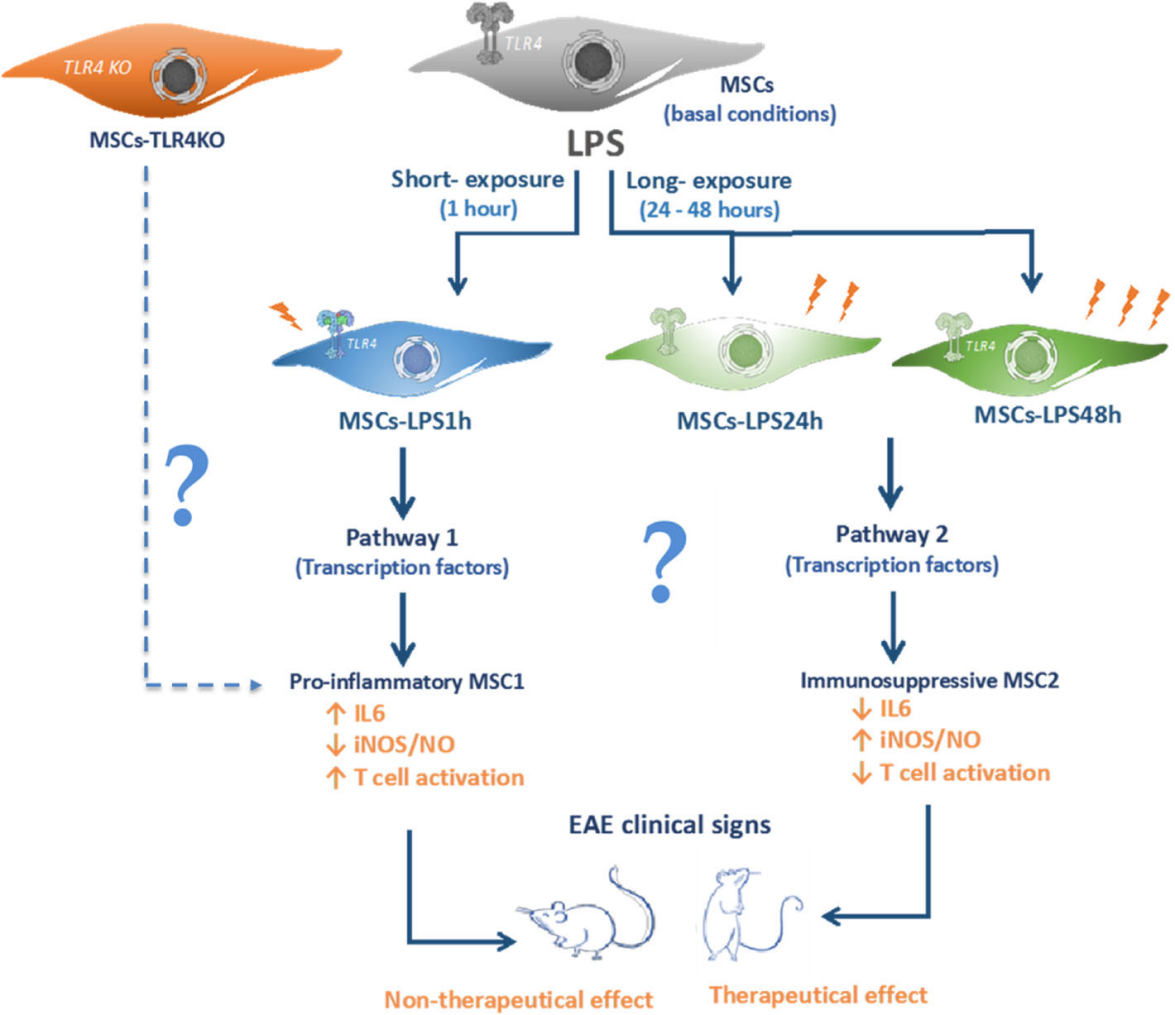
Immunoplasticity of MSCs is commanded by TLR4 expression. Schematic proposal representation of dual effect of LPS exposure in MSCs. MSCs in basal conditions express TLR4 receptor, which is activated by its ligand, LPS. Short-exposure of LPS (1 h) induce pathway 1 that leads to a pro-inflammatory MSC1 phenotype. This involves an increase in IL6 with low secretion of NO that is associated with a loss of the immune-suppressive capacity and therapeutic effect. Conversely, long-exposure of LPS (24–48 h) generates a pathway 2 that leads to a potent immune-suppressive MSC2 phenotype, increasing NO and the therapeutic potential in EAE model. MSCs-TLR4KO phenotype is similar to MSCs-LPS1h, expressing high levels of IL6, low production of NO, loss of the immunosuppressive potential in vitro, and the therapeutical effect in EAE model

Finally, we propose that MSCs undergo phenotype conversion depending on the time of LPS stimulation. Short-term stimulation could lead to a pro-inflammatory phenotype (MSC1) while longer stimulation induces an anti-inflammatory phenotype (MSC2), reflecting their high immunoplasticity (Fig. 7). This cue needs to be carefully considered in the design of clinical protocols that use MSCs in inflammatory diseases.

## Conclusions

Our results propose for the first time that MSCs can display high immunoplasticity commanded by a single stimulus mediated in part at least by TLR4. The exact molecular mechanism by which MSCs may respond differently to the same inflammatory stimuli at different exposure time remains to be deciphered. Our results underscore the importance of MSCs phenotype conversion for the design of future clinical protocols to treat patients with inflammatory and autoimmune diseases.

## Supplementary information

**Additional file 1.** Appendix A

## Data Availability

Data is available upon request to the corresponding authors.

## Abbreviations

MSCs: Mesenchymal stem cell
TLR: Toll-like receptor
LPS: Lipopolysaccharide
Poly(I:C): Polyinosinic:polycytidylic acid
IFNγ: Interferon gamma
TNF: tumor necrosis factor
IL: Interleukin
MLR: Mixed lymphocyte reaction
NO: Nitric oxide
IDO: Indoleamine 2,3-dioxygenase
TGF-β: Transforming growth factor beta
Th1: T helper 1
Th17: T helper 17
Treg: T regulatory cell
αMEM: Alpha minimal essential medium
GVHD: Graft-versus-host disease
DCs: Dendritic cells
APCs: Antigen-presenting cells
SLE: Systemic lupus erythematosus
SPF: Specific pathogen-free
EAE: Experimental autoimmune Encephalomyelitis
WT: Wild type
qPCR: Quantitative real-time PCR
FBS: Fetal bovine serum
PBS: Phosphate-buffered saline
