# Supplementary material for: Time-dependent LPS exposure commands MSC immunoplasticity through TLR4 activation leading to opposite therapeutic outcome in EAE
Source: Stem Cell Res Ther. 2020 Sep 25;11:416. doi: 10.1186/s13287-020-01840-2 (PMC7520958; doi:10.1186/s13287-020-01840-2)
Supplement: Supplementary file 1 — Additional file 1. Appendix A [file 13287_2020_1840_MOESM1_ESM.docx]

**Supplementary Data**


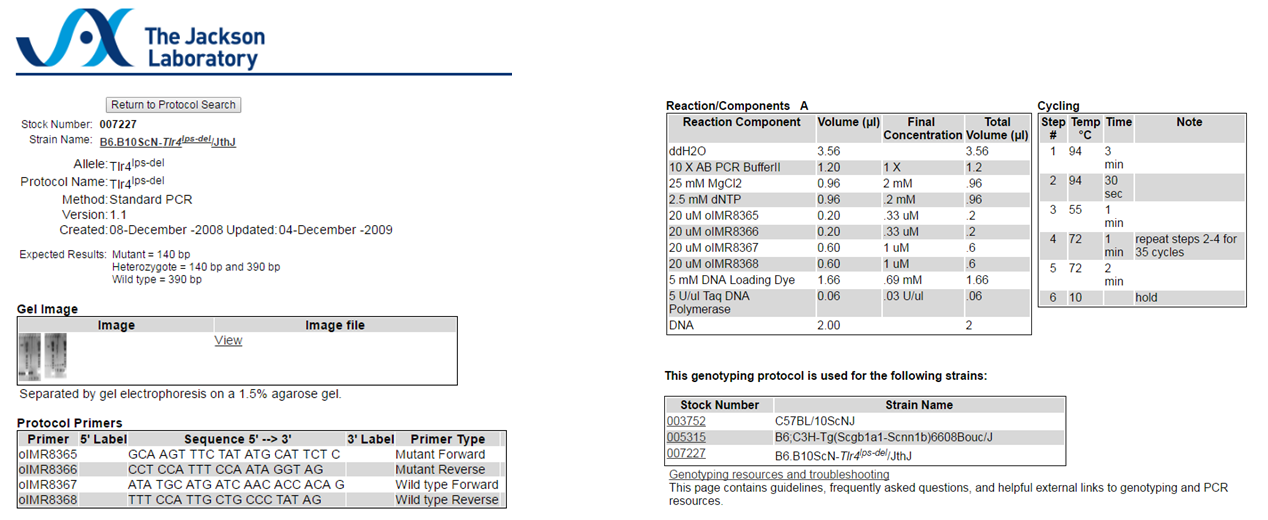


**Supplementary Figure 1.** Technical protocol used for genotypification of TLR4 mutation by PCR. Jackson Laboratory suggested using two set of primers. One of them for wild type allele and other for TLR4l^ps-del^ mutation. Expected Results: are Mutant = 140 bp, Heterozygote = 140 bp and 390 bp, Wild type = 390 bp

.

**MSCs**

**MSCs-TLR4KO**

**Supplementary Figure 2. Immunophenotype and differentiation potential of MSCs WT and MSCs-TLR4KO** **(A)** MSCs and **(B)** MSCs-TLR4KO were stained for classical murine MSC surface molecules and evaluated by FACS. Representative histogram for each antigen is shown in black line and isotype control in grey color. The capacity of MSCs to differentiate into adipocytes **(C)**, Osteocytes **(D)** and chondrocytes **(E)** was tested and evaluated by qPCR. (**F)** TLR4 expression in MSC WT (blue) and MSCs TLR4KO (red) were confirmed by genomic PCR as recommended by Jackson Laboratory. PCRs products were analyzed by agarose gel (2%) electrophoresis along with a low-range DNA ladder (MW, molecular weight). TLR4 expression was evaluated by FACS analysis in MSCs-TLR4KO (red) and MSCs wild type (Blue). Each experiment was performed at least 3 times.


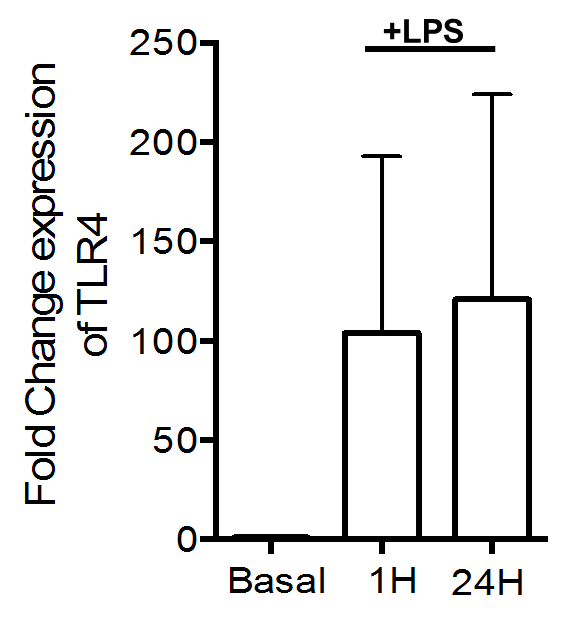


**Supplementary Figure 3: LPS activation increase the expression levels of TLR4 receptor independent on the time exposed to the ligand**. TLR4 mRNA expression levels was measured by qPCR on MSCs pretreated or not with LPS by 1 or 24 hours. Statistically analysis was performed by One-way ANOVA, Kruskal-Wallis ad-hoc post test.


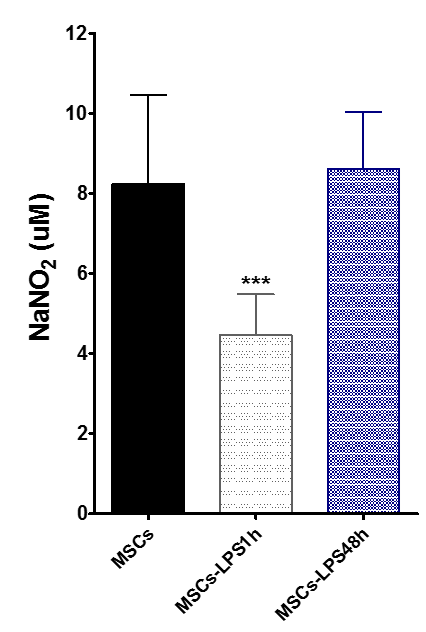


**Supplementary Figure 4: LPS activation of MSCs modulates NO production in vitro when co-cultured with activated splenocytes.** NO production was quantified in the co-culture supernatant of splenocytes with MSCs that where pretreated or not with LPS for 1 or 48 hours. Supernatants of co-cultures were collected after three days, centrifuged at 350 x g and nitric oxide (NO) was measured using a modified Griess reagent. Data are expressed as mean ± SEM; n = 3, N=3 biological replicates; ***p<0.001. Statistically analysis was performed by One-way ANOVA, Kruskal-Wallis ad-hoc post test.


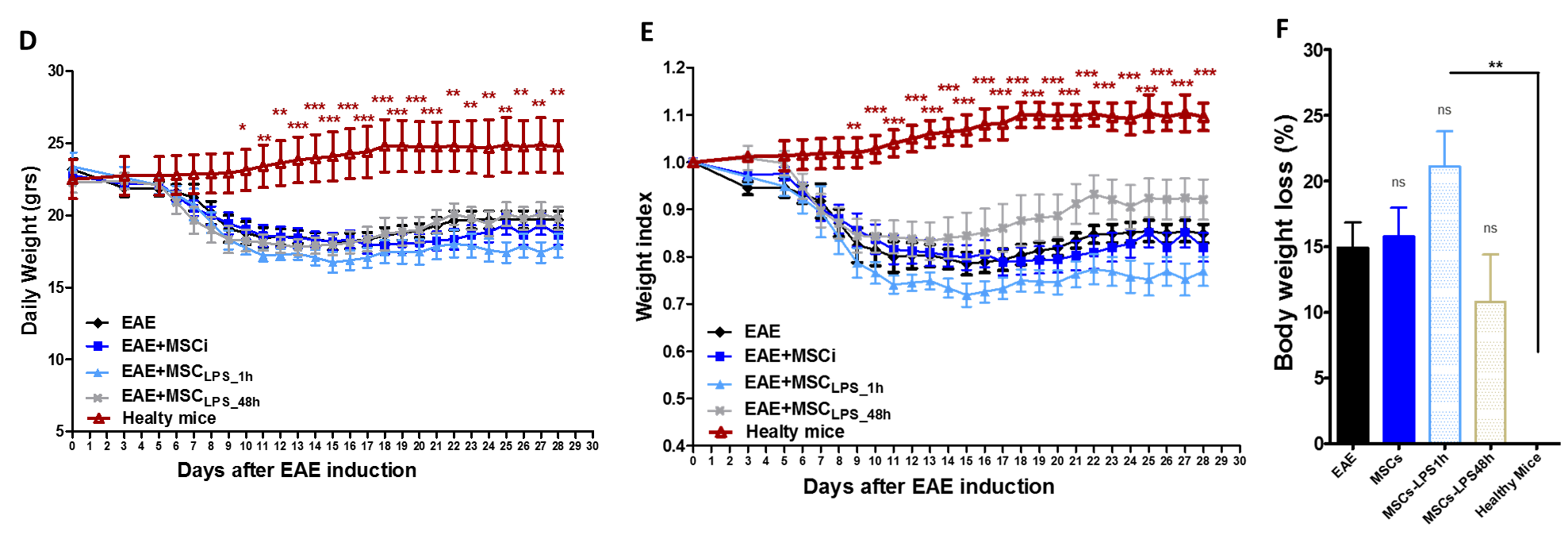

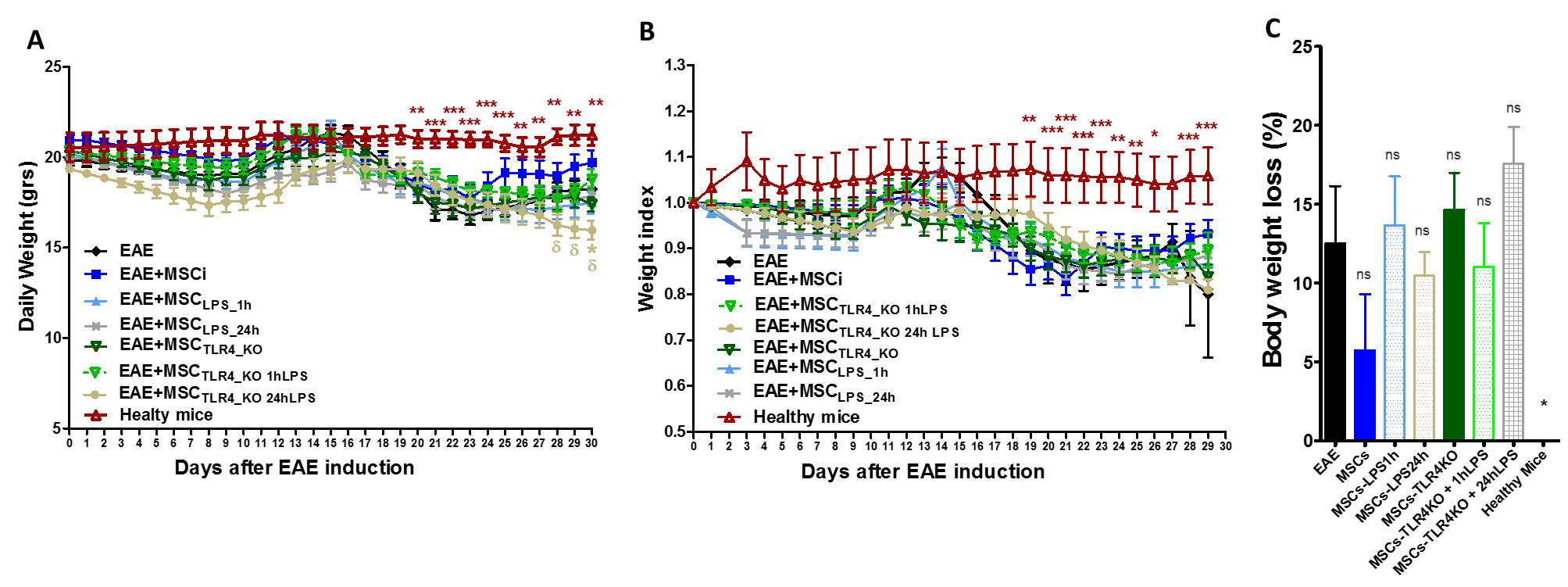


**Supplementary Figure 5. Injection of LPS-stimulated MSCs does not improve weight loss in animals with EAE.** Unstimulated MSC wt or MSCs-TLR4KO stimulated or not with LPS, (500 ng/m for 1h, 24h or 48h) were injected 7 days after EAE induction. Animal weight were evaluated daily. **(A, D)** Daily weight (grs) **(B, E)** Weight index and **(C, F)** Body weight loss (%) are shown on two independent experiments. Data are expressed as mean ± SEM; n = 8-12. * Symbol represent the comparison against EAE control group. *p<0.05. δ Symbol represents the comparison EAE+MSCs group vs. LPS treated MSCs groups. δ p<0.05. Statistically analysis was performed by Two-way ANOVA **(A, D)** and **(B, E)** or One-way ANOVA **(C, F)**.

.


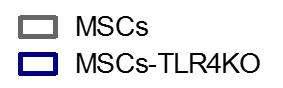


**Supplementary Figure 6: MSCs-TLR4KO produce less NO compared to MSCs WT.** MSCs WT and MSCs-TLR4KO were primed with LPS, (500 ng/ml for 24h). NO production was determined in the supernatants of MSCs using a modified Griess reagent. N=3. The results were expressed as mean ± SEM. Statistically analysis was performed by One-way ANOVA, Kruskal-Wallis ad-hoc post test.


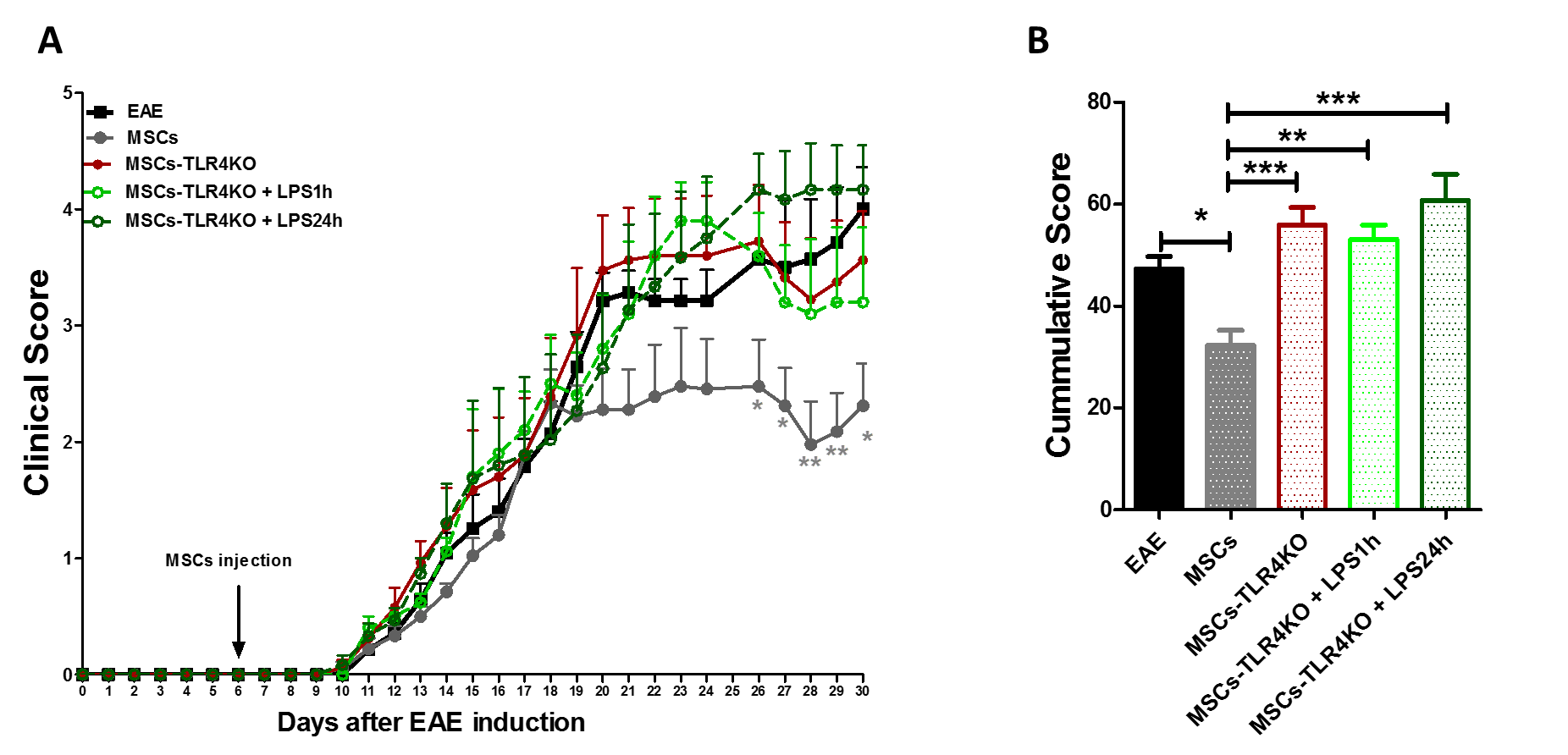


**Supplementary Figure 7.** **LPS stimulation not affect the therapeutic potential of MSCs-TLR4KO in EAE**. Unstimulated MSC wt or MSCs-TLR4KO stimulated or not with LPS, (500 ng/m for 1 or 24 h) were injected 7 days after EAE induction. (A) Daily clinical score and (B) Cumulative score. Data are expressed as mean ± SEM; n = 8-10. *p<0.05, **p<0.01, ***p<0.001. Statistically analysis was performed by Two-way ANOVA.


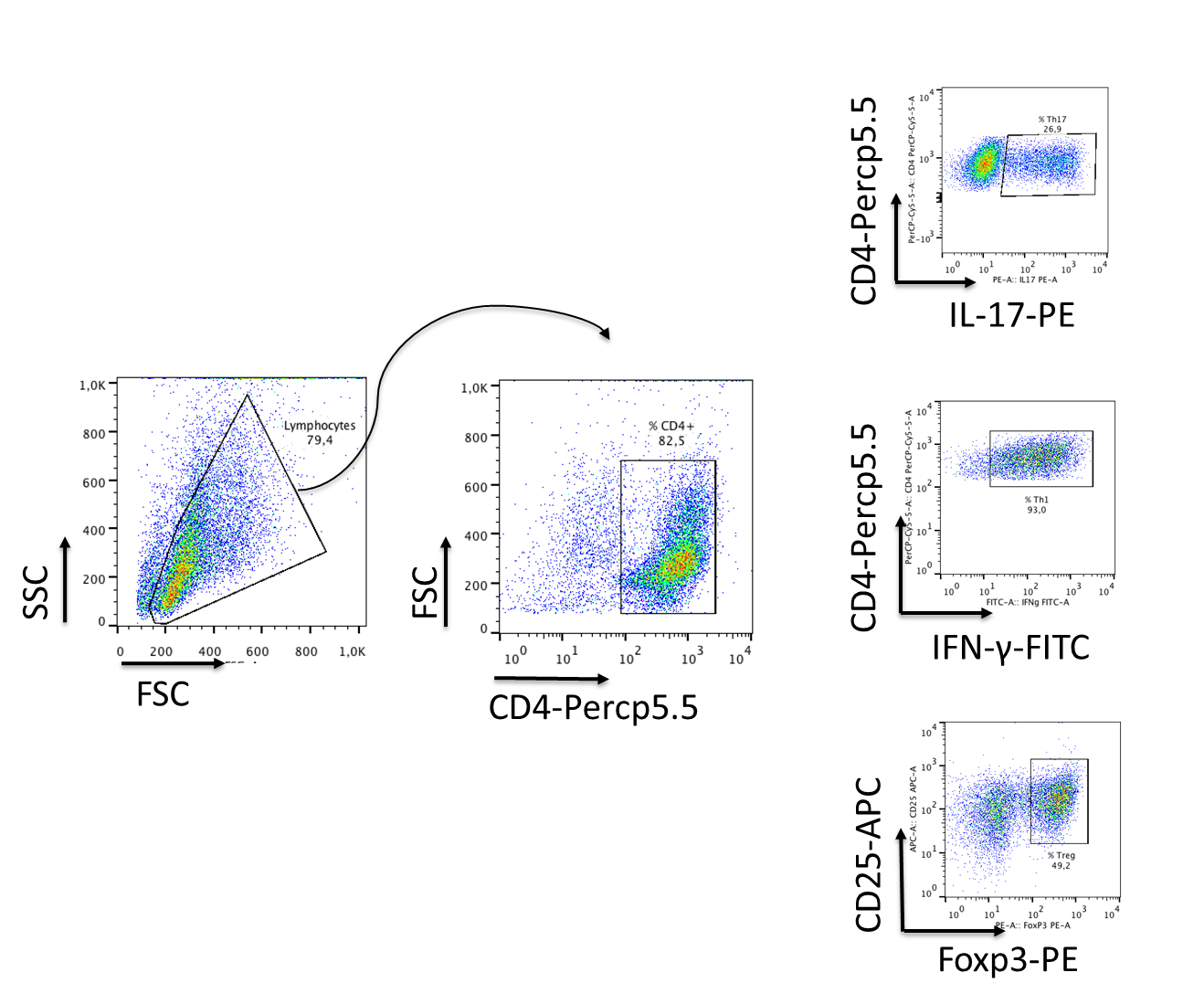


**A**

**B**

**
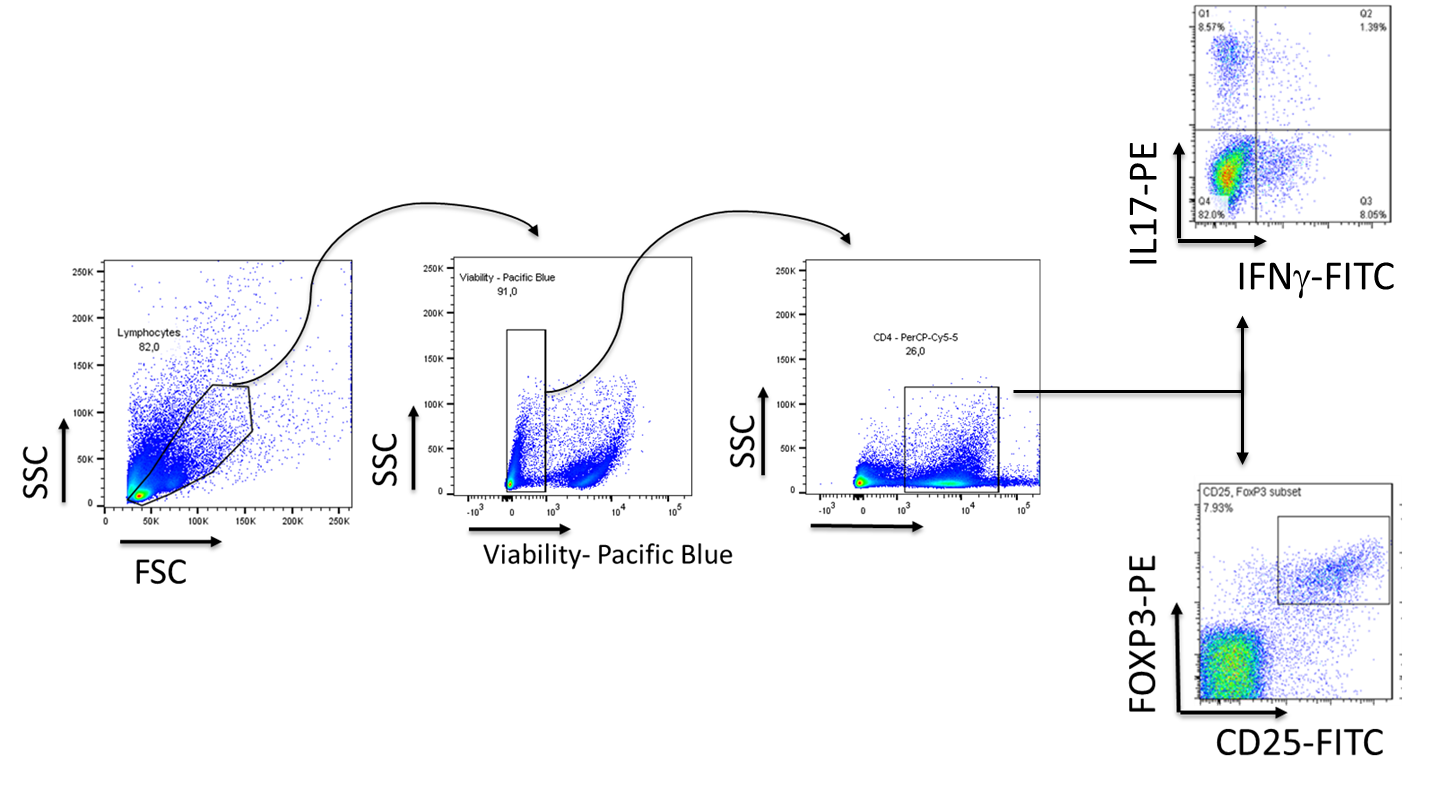
**

**Supplementary Figure 8:** Gating strategy for FACS analysis of T cell subpopulations. **(A)** in vitro differentiation of T helper Lymphocytes. **(B)** Lymph nodes of EAE animals were extracted after euthanasia and T lymphocytes were analyzed.

**Appendix A:**

**EAE Induction and MSCs Administration**.

Female C57BL/6 mice, 10 to 14 weeks old, were injected subcutaneously (s.c.) in the flank with 50 𝜇g of MOG^35–55^ peptide (LifeTein LLC, USA), emulsified in complete Freund’s adjuvant (Difco Laboratories, Detroit, MI), and supplemented with heat-inactivated Mycobacterium tuberculosis H37RA (Difco Laboratories, Detroit, MI). Two and forty-eight hours later, mice received intraperitoneally (i.p) 300 ng of Pertussis toxin (Calbiochem, La Jolla, CA). Clinical signs appeared after 10 days of EAE induction. MSCs (1 × 10^6^) diluted in PBS were i.p administrated seven days after EAE induction.

The clinical scores and mice weights were recorded daily for 30 days. Animals were evaluated according to previously published scoring scales^13^. The classical EAE scores were assigned as follows: score 0, no disease; score 0.5, reduced tail tonus; score 1, limp tail; score 1.5, limp tail and ataxia; score 2, limp tail, ataxia, and hind limb weakness; score 2.5, at least one hind limb paralyzed/weakness; score 3, both hind limbs paralyzed/weakness; score 3.5, complete paralysis of hind limbs; score 4, paralysis until hip; score 5, dead or dying animal. Animals were euthanized for ethical management if the animal have score 4 by more than 3 consecutive days or reach score 5. For scoring scales, the daily mean clinical scores and cumulative scores were calculated.
